# Diagnostic accuracy of the World Health Organization ICOPE screening tool in Brazilian older adults: a cross-sectional study

**DOI:** 10.1016/j.clinsp.2026.100915

**Published:** 2026-03-23

**Authors:** Lívia Maria do Nascimento, Thainá Gabrielle Camargo da Cruz, Juliana Fernanda de Lima e Silva, Letícia Prado Silva, Beatriz Bigatão Inácio, Marcos Eduardo Scheicher

**Affiliations:** a Graduate Program in Human Development and Technologies, Institute of Biosciences, Universidade Estadual Paulista (UNESP), Rio Claro, SP, Brazil; b Department of Physical Therapy and Occupational Therapy, Universidade Estadual Paulista (UNESP), Marília, SP, Brazil

**Keywords:** Aging, Intrinsic capacity, Screening, Integrated care

## Abstract

**Objectives:**

Evaluate the performance of diagnostic measures (sensitivity and specificity) of the Intrinsic Capacity (IC) construct by domain and by age group.

**Study design:**

164 elderly individuals were assessed in the 5 domains of IC (cognition, psychological, vitality, locomotion, and sensory) and for Activities of Daily Living (ADL), Instrumental Activities of Daily Living (IADL), frailty, and urinary incontinence. One-way ANOVA, *t*-test, or Mann-Whitney test were used for quantitative variables, and chi-square test for qualitative variables. The sensitivity and specificity were evaluated using the ROC curve.

**Results:**

Decline in IC was older, worse ADL, IADL, and mental function, low gait speed, higher depression scores, frailty, and urinary incontinence. The full version and the cognitive, psychological, locomotion, and sensory domains demonstrated moderate performance, while the vitality showed poor performance. The age-stratified analysis indicated that among participants aged 60–69 years, the cognitive, vitality, locomotion, and sensory domains showed poor performance, while the psychological domain demonstrated good performance. In the 70–79-year group, all domains showed moderate performance. In individuals aged ≥ 80-years, the cognitive, psychological, vitality, and locomotion domains demonstrated moderate performance, whereas the sensory domain showed good performance.

**Conclusions:**

Full IC construct and most domains showed moderate performance, with the exception of vitality (poor performance). When analyzed by domain and age group, performance varied across domains, ranging from poor to moderate, with the sensory domain showing the best results in the oldest group. Furthermore, the intrinsic capacity construct remains a useful screening approach for identifying older adults at risk of frailty.

## Highlights

• The study addresses a gap in IC validation for the Brazilian elderly.
• Age-stratified analysis tailors screening to high-risk groups.
• ADL, IADL, frailty, and urinary incontinence enrich the IC evaluation.

## Introduction

The world population is aging rapidly, with projections indicating that by 2050, there will be 2.1 billion people over the age of 65 in the world.^1^ In Brazil, demographic data suggests that the number of elderly people will increase from 32 million in 2023 to 66 million in 2050,^2^ indicating that additional spending will be necessary.^3^

Faced with the significant increase in the population aged over 60, the World Health Organization (WHO) published the World Report on Ageing and Health in 2015, reformulating and reorienting global actions aimed at healthy ageing.^4^ Aging healthily involves maintaining and developing functional capacity and intrinsic capacity, promoting well-being in old age, even in the presence of diseases.^5^ The concept of Integrated Care for Older People (ICOPE) then emerged, focusing on maintaining the skills and abilities of the elderly and not just on the losses/illnesses of aging.^6^^,^^7^

Intrinsic Capacity (IC) was defined as the set of physical and mental capacities that a person can use throughout their life and its construct was developed based on five essential, but not unique, domains: cognitive, psychological, locomotor, vitality and sensory.^8^ Through the CI and its domains, it is possible to carry out monitoring that is contextualized with the specificities of the individual and the population analyzed, as well as to evaluate the effectiveness of interventions already carried out or to indicate interventions that are needed in the short term (red flags).^9^^,^^10^ One of ICOPE's ideas is longitudinal screening of the IC domains, checking whether the person is out of expectations and thus has time for correction.

Despite being a valuable and person-centered instrument and efforts being made to implement it in several countries, there is little evidence on the clinical usefulness of the ICOPE tool,^11^^,^^12^ requiring validation studies of the instrument and studies on how to incorporate it into care models.^13^ In addition, evidence on its feasibility, diagnostic accuracy, psychometric characteristics and real utility is still scarce.

The WHO points out that intrinsic capacity is expected to decrease with age,^7^ suggesting the importance of its assessment by age group. Furthermore, it seems clear that, as opposed to evaluating the construct as a whole,^11^^,^^12^^,^^14^^,^^15^ it is better to evaluate each domain separately,^13^^,^^16^ so that possible interventions/monitoring are individualized for each domain.

Therefore, the objective of the study was to evaluate the performance of diagnostic measures (sensitivity and specificity) of the intrinsic capacity construct by domain and by age group.

## Material and methods

### Study design and participants

The authors followed the STARD guidelines (Standards for Reporting of Diagnostic Accuracy Studies). It was a cross-sectional study that included elderly people living in the coverage area of two Family Health Units (USFs in Portuguese acronym) in the city of Marília, São Paulo, Brazil. A total of 1113 older adults are registered with the two USFs. The study was approved by the research ethics committee of the Faculty of Philosophy and Sciences-Unesp/Marília (protocol n° 4168,934). The eligibility criteria were: ≥ 60-years-old, not having diagnosed dementia, not having acute heart failure, infection, cerebrovascular disease, severe cardiac, hepatic, or renal dysfunction, Parkinson's disease, blindness and deafness. All eligible participants or their companions signed an informed consent form after reading it. For vulnerable participants, the study procedures were explained to their accompanying persons, who then provided written informed consent. The sample size was calculated with a power of 80% and an alpha error probability of 0.05, with a loss prevalence of 10%, resulting in a total of 176 individuals. The final sample evaluated included 164 older people, with the loss of twelve participants (6.8%) due to incomplete questionnaires. The missing data were treated using the complete-case analysis method,^17^^,^^18^ with all individuals in this situation excluded from the analysis. Even though sample loss may mean a decrease in the representativeness of the sample, the power of the final sample (164 cases) remained high (0.98), with minimal risk of bias. Sensitivity analyses ‒ including recalculation of statistical power, comparison of baseline characteristics between included participants and losses, and extreme-case simulations ‒ showed that this small loss did not change the magnitude or direction of the estimated effects, indicating that the findings are robust to sample loss. In relation to the total sample evaluated (164), the power of each age group was: 0.97 for >80-years and 0.99 for 60‒69 and 70‒79-years.

A search of individuals aged 60-years or over who were registered with the USFs was performed through the e-SUS Primary Care digital platform. Next, the assessment was scheduled in two ways: 1) The older adults were contacted by telephone and invited to come to the USF to participate in the study; 2) Community agents of the USFs visited the homes of the older adults and performed the assessments on those who agreed to participate.

### Assessments

#### Domains

The evaluations were carried out by previously trained researchers. The method suggested by Beard et al.^19^ was adapted, evaluating the five proposed domains as follows:

**Cognitive domain:** The Montreal Cognitive Assessment (MoCA) was used to assess the participants' cognitive domain. The total test score is 30-points and normality scores vary according to the level of education.^20^
**Psychological Domain:** The 15-item Geriatric Depression Scale (GDS-15) was used to analyze the psychological domain. Its advantages include easy-to-understand questions, small variation in the possible answers (yes/no), and self-administration or administration by an interviewer. The score ranges from 0 (absence of depressive symptoms) to 15 points (maximum score of depressive symptoms).
**Vitality Domain:** The Mini Nutritional Assessment (MNA), a tool recommended by the WHO,^21^ was used to assess this domain, with scores ranging from 0 to 14: ≥ 12 indicates a satisfactory nutritional status; between 8 and 11 indicates a risk of malnutrition; and a score <8 indicates malnutrition.^22^
**Locomotion Domain:** Short Physical Performance Battery (SPPB). The SPPB is a tool for assessing lower limb function through a battery of static balance tests, gait speed and the test of getting up from a chair five consecutive times (lower limb strength). Each test can be evaluated separately or together, generating a total score of 0 (worst performance) to 12-points (best performance).^23^
**Sensory Domain:** Hearing and visual impairments were assessed using a self-report with previously validated questions.^24^^,^^25^ Hearing status was assessed by asking participants to rate their hearing as excellent, very good, good, fair or poor (with the help of a hearing aid, if they wore one). For vision, participants were also asked “how good is your vision for seeing things at a distance, such as recognizing a friend across the street” and “how good is your vision for seeing things up close, such as reading a newspaper”, categorizing the answer options as excellent, very good, good, fair or poor. This assessment was made with the participant wearing glasses or corrective lenses, if they normally did so. For each answer given in relation to hearing and vision, a score was assigned according to the Likert scale: 1 - Poor, 2 - Fair, 3 - Good, 4 - Very good and 5 - Excellent. An average was taken of the three assessments (far vision, near vision and hearing) to determine the scores for this domain.

#### Secondary outcomes

**Level of dependence:** The level of dependence can interfere with the domains of intrinsic capacity. For this reason, the Activity of Daily Living (ADL) and Instrumental Activity of Daily Living (IADL) were also assessed. The Barthel Index was used to assess ADL and the Lawton scale for IADL.
**Urinary incontinence:** Urinary Incontinence (UI), defined as any involuntary loss of urine,^26^ can negatively impact lifestyle, limiting daily and social activities. As UI is considered a geriatric syndrome^27^ and can also interfere in the domains of intrinsic capacity, complaints related to urinary incontinence were assessed using the ICIC-SF.
**Frailty:** Frailty is the result of organic deficiencies, which generate alterations in the healthy ageing process. Research has shown that the level of frailty can improve or worsen over time.^28^^,^^29^ In this study, frailty was assessed using the Edmonton Frail Scale (EFS).^30^

#### Other evaluations

The following variables were considered in the possible explanation of the outcomes: age, gender, schooling and housing conditions.

### Statistical analysis

Descriptive statistics were used to summarize the intrinsic capacity and identify which domains were compromised. Comparisons between three or more samples were made by 1-way ANOVA with Bonferroni post-test and those with two samples by the *t*-test or Mann-Whitney test for quantitative variables. Chi-Square test was used for qualitative variables. Spearman correlation coefficients were calculated to assess the relationship between IC and EFS scores. All analyses were performed using SPSS 20.0.

Each domain of intrinsic capacity received a score of zero when it was in disagreement with the reference values of the tests applied: Cognitive domain (MOCA): according to education (illiterate: ≤ 11; 1- to 4-years of study: ≤ 16; 5- to 11-years of study: ≤ 19; ≥ 12-years of study: ≤ 25);[20] locomotion domain (SPPB) ≤ 9; psychological domain: > 5; vitality domain (MNA) ≤ 11; sensory domain: ≤ 2 (Likert scale ranging from 0 to 5).

The total intrinsic capacity score ranged from zero to five, with higher scores indicating better intrinsic capacity. Values between zero and two were indicated as having a decline and values between three and five as not having a decline.

The sensitivity and specificity of each domain and each domain by age group were evaluated using the ROC curve. A screening tool is considered to have good performance if sensitivity and specificity are ≥ 80%; reasonable performance, if sensitivity or specificity are < 80%, but both values ≥ 50%; and poor performance if sensitivity or specificity is < 50%. In addition, the Youden index was also used, which summarizes the sensitivity and specificity of a tool. A Youden index of 1 indicates perfect sensitivity and specificity, and a value of 0 indicates that the test is not useful. The ROC curve, Youden index and cut-off values were determined using Medcalc software.

Sensitivity analyses were conducted to assess the stability of domain-specific performance across small age-based subgroups (60–69, 70–79, ≥ 80-years). For each subgroup, the AUC and its 95% CI were examined, using CI width (Upper-lower) as an indicator of precision. Subgroups were considered potentially unstable when the CI width exceeded 0.25 or when the upper CI limit reached 1.00.

## Results

The mean IC score was 2.73 ± 1.33. Of the total number of participants, only 17 (10.4%) were categorized as not having declined in any domain. On the other hand, of the 164 elderly people included, 40 (24.4%), 43 (26.2%), 41 (25%), 18 (11%) and 5 (3%) showed a decline in one, two, three, four or five domains, respectively. The percentage of people with a decline in each domain was: Cognitive: 40.8%; Psychological: 9.7%; Vitality: 43.3%; Locomotor: 63.4%, Sensory: 53.6%.

**Fig. 1:**
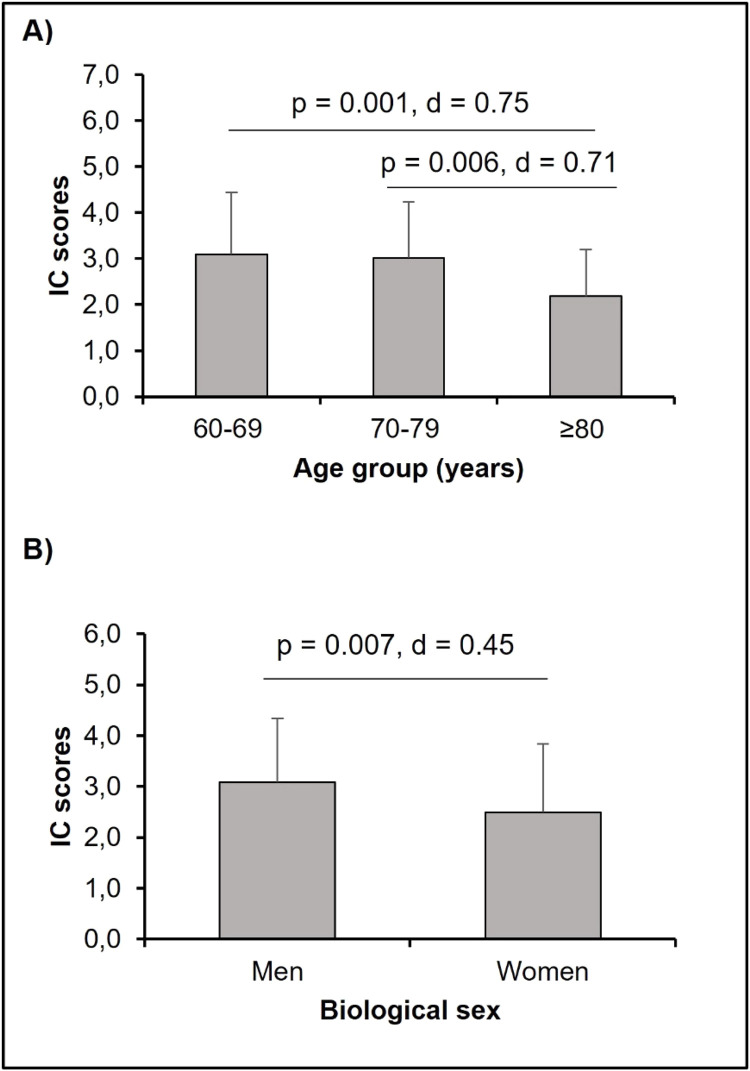
Comparisons of IC scores by age groups and biological sex.

**Fig. 2:**
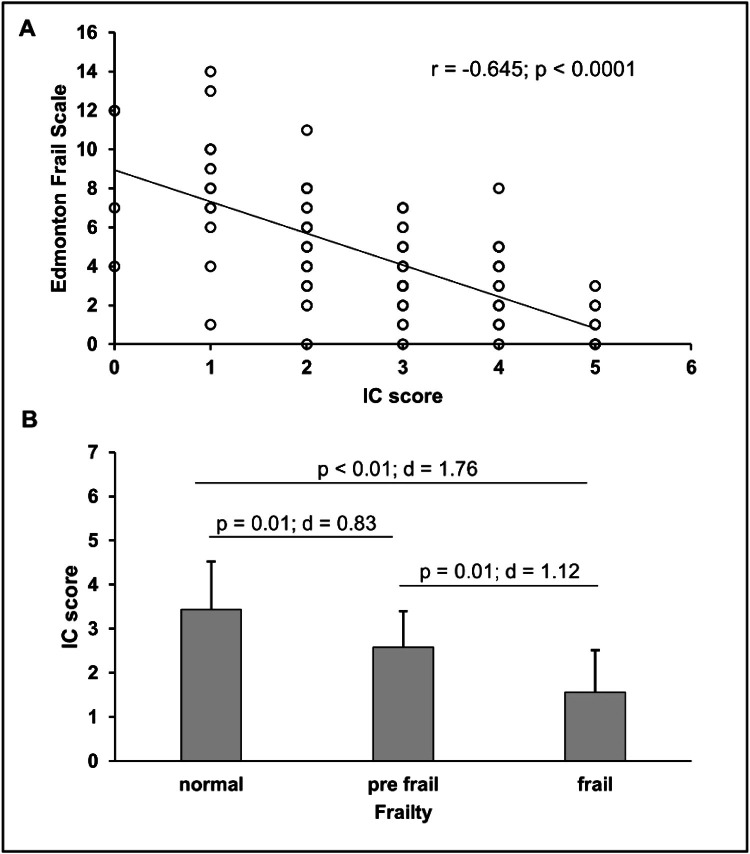
(A) Correlation between IC scores and Edmonton Frail Scale; (B) Comparison of intrinsic capacity score among robust, prefrailty, and frailty, assessed by Edmonton Frail Scale.

The present data showed an effect of age on IC scores ([2.0.161] = 6.574; *p* = 0.002) with a significant difference between the 65‒69 and ≥ 80 age groups (*p* = 0.002) and between the 70‒79 and ≥ 80 age groups (*p* = 0.006), reinforcing the importance of its assessment by age group, especially in the over-80 s. There was also a difference between men and women (3.1 ± 1.26 and 2.5 ± 1.35, respectively). The comparisons of IC scores by age group and biological sex can be seen in Fig. 1. Fig. 2 shows the comparisons of IC scores according to the Edmonton Frail Scale classification and the correlation between the EFS and IC scores. There was a significant difference and a large effect size between the intrinsic capacity scores according to frailty. In addition, the data showed a negative correlation between the variables. In Table 1, it is possible to observe that older people with a decline in IC scores had a significant difference in EFS values compared to those without a decline.

**Table 1:** Characteristics of participants with and without a decline in intrinsic capacity.

	Non-DIC (*n* = 100)	DIC (*n* = 64)	χ^2^	Cramer's V	p-value
**Age group (years)**	70.66±7003	75.46±7.86			0.0003
**Biological sex, n (%)**					
Male	48 (69.6)	21 (30.4)	3.20	0.14	0.07
Female	53 (55.8)	42 (44,2)			
**Gait speed (m/s)**	0.93±0.24	0.71±0.27			<0.000
**Postural balance (SPPB)**	9.60±1.74	6.39±3.02			0.000
**IADL, n (%)**					
Dependent	25 (39.1)	39 (60.9)	30.21	0.43	0.003
Non-dependent	76 76.0)	24 (24.0)			
**ADL, n (%)**					
Dependent	12 (35.3)	22 (64.7)	20.99	0.36	0.004
Non-dependent	89 (68.5)	41 (31.5)			
**Education level, n (%)**					
Incomplete primary education	55 (56.1)	43 (43.9)	12.67	0.28	0.01
Complete primary education	21 (58.3)	15 (41.7)			
Complete high school	17 (94.4)	1 (5,6)			
Higher education	3 (50.0)	3 (50.0)			
Postgraduate	5 (83.3)	1 (16.7)			
**Frailty, n (%)**					
Robust	84 (81.6)	19 (18.4)	52.06		0.000
Prefrail	11 (45.8)	13 (54.2)		0.56	
Frail	6 (16.2)	31 (86.8)			
**Edmonton Frail Scale**	3.20 ± 1.89	6.44 ± 2.99			0.000
**Urinary incontinence, n (%)**					
Yes	23 (41.9)	32 (58.1)	12.85	0.28	0.000
No	78 (71.6)	31 (28.4)			
**Physical activity, n (%)**					
Yes	43 (93.5)	3 (6.5)	24.27	0.38	0.000
No	58 (49.2)	60 (50.8)			
**Living, n (%)**					
Alone	17 (65.4)	9 (34.6)	0.09	0.02	0.76
Accompanied	84 (60.9)	54 (39.1)			
**MoCa**^a^	20.77±5.03	15.75±6.59			<0.000
**GDS**	3.51±2.29	5.48±3.32			<0.000

Data were expressed as mean ± standard deviation or n (%); DIC, Decline in Intrinsic Capacity; Non-DIC, No Decline in Intrinsic Capacity; MoCa, Montreal Cognitive Assessment; GDS, Geriatric Depression Scale; IADL, Instrumental Activity of Daily Living; ADL, Activity of Daily Living; m/s, Metes per Second.

a Adjusted by schooling.

As shown in Table 1, participants with declines in IC were older, had worse ADL function and low gait speed. They had worse mental function, indicated by lower MoCa scores, and had higher GDS scores. In addition, people with a decline in IC had frailty, urinary incontinence, and worse IADL activities. Other issues related to the decline in CI were self-reported non-practice of physical activities and level of schooling. Table 1 also shows the association (Cramer's V) between some variables and the decline in IC.^31^ There is a very small association in the living variable; a small association in the schooling variable; a moderate association in the ADL, IADL, and physical activity variables and a large association in the frailty variable.

**Fig. 3:**
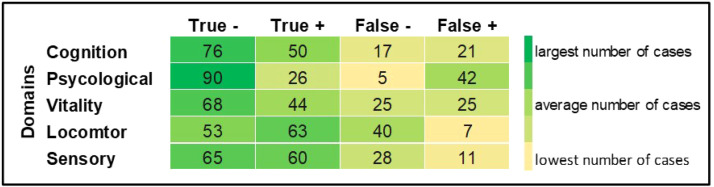
Heatmap showing the number of cases by domain.

Table 2 shows the ROC curve and Youden index values for the domains separately, while Fig. 3 shows the distribution of cases in each domain.

**Table 2:** Sensitivity, specificity and AUC-ROC of the intrinsic capacity domains.

	Sensitivity (%)	Specificity (%)	AUC	95% CI	p	Youdem index
**Full assessment**	59.0	79.0	0.75	0.758–0.816	<0.001	0.42
**Domains**						
Cognitive	79.4	73.1	0.83	0.768–0.887	<0.001	0.52
Psychological	68.2	83.9	0.86	0.799–0.911	<0.001	0.52
Vitality	49.5	88.4	0.77	0.699–0.834	<0.001	0.37
Locomotion	93.3	57.3	0.83	0.770–0.889	<0.001	0.50
Sensory	68.4	94.3	0.87	0.808–0.917	<0.001	0.62

AUC, Area Under Curve; CI, Confidence Interval.

**Fig. 4:**
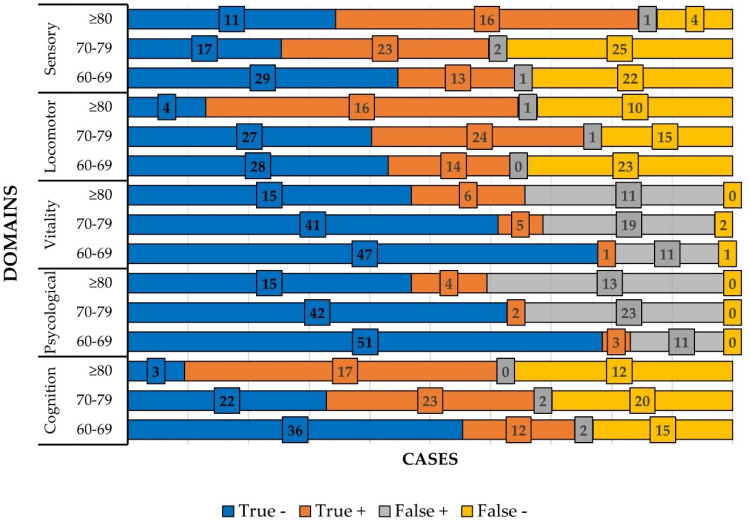
Number of cases in each domain, by age group (in years).

Table 3 shows the ROC curve and Youden index values for each domain by age group and Fig. 4 shows the absolute frequency in each domain by age group. Several subgroups demonstrated reduced precision, reflected by wide CIs or upper CI limits of 1.00 ‒ findings consistent with small subgroup sizes. These included: Cognitive (≥ 80-years), Psychological (60–69 and 70–79-years), Vitality (all age groups, especially 60–69-years), Locomotion (≥ 80-years), and Sensory (≥ 80-years). Although point estimates (AUCs) were generally acceptable, the imprecision observed in these strata indicates that these results should be interpreted cautiously.

**Table 3:** Sensitivity, specificity, AUC-ROC and Youden index of the intrinsic capacity domains by age group.

		Age group (years)	Sensitivity (%)	Specificity (%)	AUC	95% CI	p	Youdem index
**Domains**	**Cognitive**	60–69	44.4	94.7	0.812	0.714–0.910	<0.001	0.39
		70–79	53.5	91.7	0.817	0.718–0.917	<0.001	0.45
		≥80	100	58.6	0.845	0.690–1.00	<0.001	0.58
	**Psychological**	60–69	100	82.3	0.935	0.863–1.00	<0.001	0.82
		70–79	100	64.6	0.904	0.727–1.00	<0.001	0.65
		≥80	100	53.6	0.964	0.912–1.00	<0.001	0.54
	**Vitality**	60–69	20	80	0.657	0.433–0.881	0.17	0.01
		70–79	71.4	68.3	0.754	0.599–0.908	0.001	0.40
		≥80	100	57.7	0.949	0.868–1.00	<0001	0.58
	**Locomotion**	60–69	37.8	100	0.835	0.749–0.922	<0.001	0.38
		77–79	61.5	96.4	0.832	0.742–0.922	<0.001	0.58
		≥80	61.5	80	0.754	0.551–0.956	0.014	0.42
	**Sensory**	60–69	37.1	96.7	0.841	0.752–0.930	<0.001	0.34
		70–79	47.9	89.5	0.821	0.708–0.935	<0001	0.34
		≥80	80	91.7	0.854	0.708–1.00	<0.001	0.72

AUC, Area Under Curve; CI, Confidence Interval.

Table 4 shows the values of the ROC curve that was performed to explore the performance of the intrinsic capacity tool in relation to frailty, ADL and IADL. It is important to note that the instrument performed moderately well for frailty and poorly for ADL and IADL.

**Table 4:** The estimating sensitivity, specificity, AUC-ROC and Youden index of the ICOPE screening tool.

	Sensitivity (%)	Especificity (%)	AUC	95% CI	p	Youden index
**Frailty**	100	78.7	0.89	0.671–0.810	<0.001	0.78
**IADL**	65.6	78	0.71	0.673–0.811	<0.001	0.43
**ADL**	82.4	72.3	0.77	0.665–0.804	<0.001	0.54

AUC, Area Under the Curve; ICOPE, Integrated Care fou Older Pepple; IADL, Instrumental Activities of Daily Living; ADL, Activities of Daily Living; CI, Confidence Interval.

## Discussion

The population is getting older in an unprecedented phenomenon in the history of humanity. Because of this, the WHO launched in 2017 a person-centered care model called Integrated Care for Older People (ICOPE), based on functional ability and intrinsic capacity. Through the construct of intrinsic capacity, it is possible not only to indicate necessary treatments, but also to prevent diseases from occurring. Despite the importance of the subject, there is little evidence of the validity of this construct in the literature.^13^

The present results showed that in the population studied, 89.6% of people showed one or more declines in IC, a percentage above that found in other studies[11,12] and close to the research carried out by Tavassoli et al. - 94.3%.^32^ As in other research, the data also showed a decline in IC with advancing age, in line with the intrinsic capacity model formulated by the WHO.^8^^,^^11^^,^^12^

Another finding was that men had higher IC scores than women (Fig. 1). However, the fact of being a woman or a man is not implicated in the development of IC decline (Table 1). The comparisons made between the groups showed that the older people who had a decline in intrinsic capacity performed worse in postural balance, cognition, and psychological aspects. This indicates that these people are not aging healthily.

From a healthy aging perspective, assessing frailty and/or intrinsic capacity can help understand older adults' priorities. Frailty is defined as an age-related, multi-causal syndrome that negatively affects the homeostatic reserves of older adults, predisposing them to a high risk of negative outcomes.^33^ The present data, such as in another study,^12^ showed that elderly people with a decline in IC are more prone to developing frailty. A longitudinal study by Liu et al.^34^ followed the trajectory of IC and frailty in older people living in the community and concluded that IC impairment and frailty overlap and coexist in older adults, and that it is important to observe both issues in order to detect early declines in intrinsic capacity and/or the presence of frailty. Despite being a cross-sectional study, the present data points in the same direction, as there was a correlation between IC scores and EFS scores. With regard to the diagnostic measure of frailty, with a cut-off ≤4, the CI tool performed moderately well and could be used as a screening tool to identify people at risk of frailty. In another study, Belloni and Cesari stated that intrinsic capacity and frailty have similarities and peculiar points and that the IC construct can be considered as a sort of evolution of frailty, in the sense that the authors have to look at the capacities that are still preserved and not just the losses.^33^ However, there is another issue to discuss. Is an elderly person with a decline in intrinsic capacity more likely to develop frailty, or does being a frail elderly person cause them to have a decline in intrinsic capacity? Where does each syndrome start? In fact, the health of the older person should be monitored at all times and not just when they have disabilities. In Table 4, using a cutoff of 3, it was shown that IC was effective in identifying older adults with frailty. In another study, Ma et al. also found good effectiveness in identifying frail elderly people using the ICOPE tool.^12^

Regarding diagnostic measures, when the assessment was made in relation to each domain, none of them proved to be a good screening tool (sensitivity and specificity > 80%), but four of them can be classified as a moderate screening tool (sensitivity or specificity 〈 80%, but both values 〉 50%), with the exception of vitality (poor screening tool). The Youden index also performed reasonably well. The full assessment showed a bad performance with AUC < 0.8 with sensitivity and specificity < 0.8 too. Clinically, it seems to be better to assess each domain separately rather than all together.

When the analyses were made by domain and age group, the following was found: cognitive domain with moderate performance for all age groups; good performance in the psychological domain in the 60‒69 and ≥ 80 age groups and moderate performance for those aged 70‒79; poor performance in the vitality domain in the 60‒69 and 70‒79 age groups and moderate performance in those aged ≥ 80; in the locomotion domain, moderate performance was observed in all age groups; in the sensory domain, moderate performance was observed in the 60‒69 and 70‒79 age groups and good performance in the ≥ 80 age group. Clinically and in comparison with the other assessments carried out, it seems preferable to use analysis by domain and by age group, especially if the person is over 80-years-old.

Only one study validating intrinsic capacity in the Brazilian population was found.^32^ This study concluded that the IC construct is valid and reliable for assessing healthy ageing in diverse socioeconomic and cultural settings. The present data showed that it is possible to apply the IC construct in the evaluation of the elderly population, with good sensitivity and specificity, but it should be noted that the evaluation should be separated by domain and by age group.

The IC construct proposed by WHO should be used as a screening tool for assessing the presence/decrease/absence of capabilities. With this screening, it is possible to determine the individuals most at risk of developing/having alterations (red flags) and those who can be monitored over time. For red flags, it will be possible to determine better treatments and the responses to them. This data showed that the majority of the population evaluated had one or more domains with decline. This means that urgent action is needed to ensure that these elderly people do not experience a deterioration in their abilities, but have the possibility of healthy ageing.

Given these issues, the question arises: how could the Brazilian public health system use/implement the ICOPE screening instrument in a country of continental dimensions, with so many sociodemographic variations and whose public health system is basically curative rather than preventive? It would be a slow process involving the following steps: 1) Incorporation into Primary Health Care (PHC), integrating ICOPE into Family Health Strategy routines; 2) Professional training, preparing teams to apply the model’s steps in a standardized way; 3) Functional screening, identifying early declines in intrinsic capacity domains; 4) Multidimensional clinical assessment when screening indicates alterations; 5) Development of personalized interventions based on functional needs; 6) Periodic monitoring and adjustment of the care plan according to progress; 7) Use of platforms such as ICOPE Monitor for recording and follow-up; 8) Network integration, coordinating PHC, specialized services, and social assistance for comprehensive care. The ideal would be a national survey coordinated by the Ministry of Health in partnership with research centers, or study protocols as proposed by Ferrioli et al.^35^ It's not easy, but it is possible.

The study had some limitations: 1) It is not possible to extrapolate the results to the population living in rural areas, as well as to those living in long-term care facilities, due to the characteristics of these individuals. Further studies with these populations are needed, using the concept of intrinsic capacity; 2) It was conducted in a single location, and more research is needed to carry out future multicenter studies with stratified sampling to increase external validity; 3) The cross-sectional design does not allow for establishing causality or long-term trends in intrinsic capacity, and longitudinal follow-up studies should be conducted to assess IC trajectories; 4) Limitations of small subgroups: several age ranges showed low precision attributable to the small sample sizes. Therefore, the results from the subgroups should be considered with caution.

### Clinical and practical implications

**Fig. 5:**
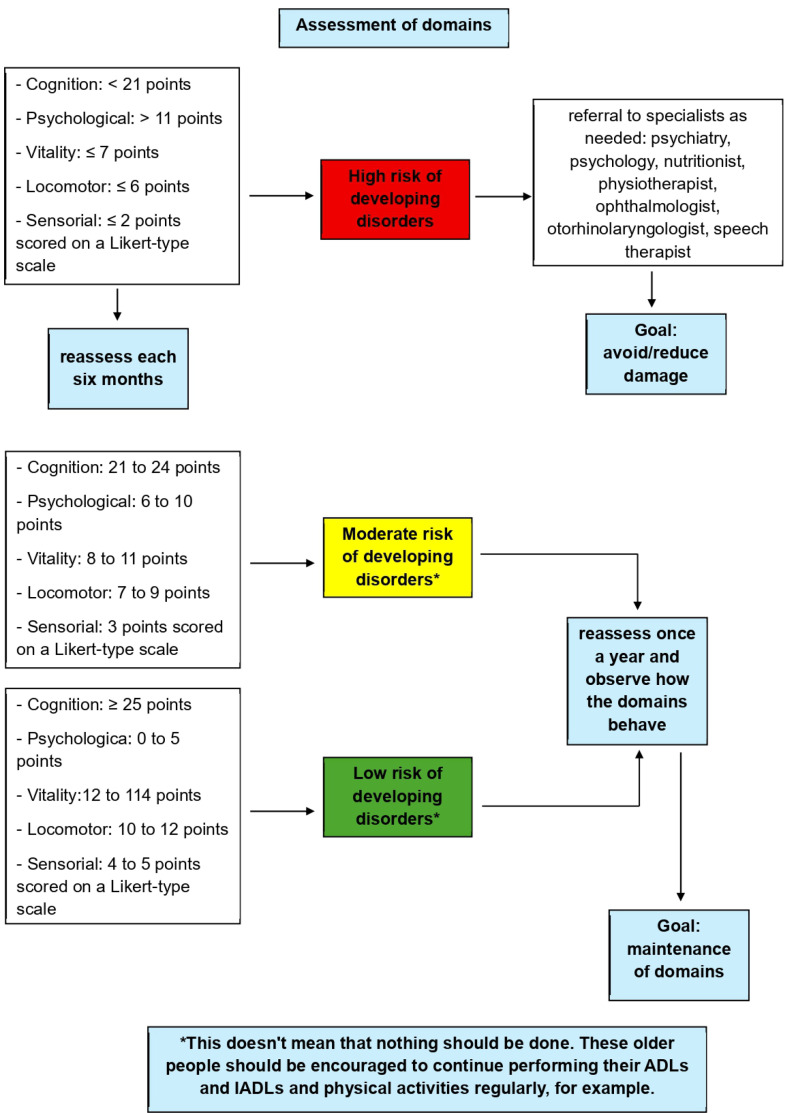
Algorithm for assessment, clinical decision and monitoring of intrinsic capacity domains.

Screening a specific population is a crucial tool for understanding their short-, medium-, and long-term needs. Fig. 5 allows healthcare professionals to visualize the cut-off points for each instrument used across the five areas assessed within the intrinsic capacity construct. In addition, the figure makes it easier for clinicians to identify the risk of older adults developing disorders ‒ high, moderate, or low ‒ and describes the recommended referrals and reassessment intervals for high-risk individuals, as well as the appropriate actions and follow-up schedules for those at moderate or low risk.

For example, the study showed that 63.4% of the elderly individuals evaluated presented changes in the locomotion domain. Therefore, it is necessary to classify the risk of falls in these elderly individuals according to the test cut-off score (≤ 6-points indicates high risk, 7‒9 points indicates moderate risk, and 10‒12 points indicates low risk for SPPB – Fig. 5). Individuals classified as high risk should be referred to a specialized professional, such as a physical therapist, for a comprehensive assessment to determine the most appropriate interventions aimed at improving postural control and, consequently, postural balance. An equivalent procedure must be applied to the remaining domains as well (Fig. 5). Another clinical application is that the intrinsic capacity has been influenced by age, with older adults having lower scores than the other age groups. Another important clinical issue is that the tool can be used to identify older people at risk of frailty.

Most importantly, unlike traditional disease-based metrics, Intrinsic Capacity (IC) offers a continuous assessment of core functional domains that capture what older adults value most: the preservation of independence, vitality, and quality of life.^36^

## Conclusions

The intrinsic capacity construct showed moderate performance when all domains were used simultaneously as a single tool. When the application was separated by domain, performance was moderate for most domains, and by domain and age group, performance ranged from poor to moderate, with the sensory domain showing the best results in the oldest group. Furthermore, the construct can be used as a screening tool to identify people at risk of frailty.

## Data availability

The datasets generated and/or analyzed during the current study are available from the corresponding author upon reasonable request.

## Funding

This research did not receive any specific grant from funding agencies in the public, commercial, or not-for-profit sectors.

## Declaration of competing interest

The authors declare no conflicts of interest.
